# A Lactose-Binding Lectin from the Marine Sponge *Cinachyrella Apion* (Cal) Induces Cell Death in Human Cervical Adenocarcinoma Cells

**DOI:** 10.3390/md10040727

**Published:** 2012-03-28

**Authors:** Luciana Rabelo, Norberto Monteiro, Raphael Serquiz, Paula Santos, Ruth Oliveira, Adeliana Oliveira, Hugo Rocha, Ana Heloneida Morais, Adriana Uchoa, Elizeu Santos

**Affiliations:** 1 Department of Biochemistry, Federal University of Rio Grande do Norte, Natal, RN, 59072-970, Brazil; Email: luciana_marabelo@hotmail.com (L.R.); norbertokv@gmail.com (N.M.); raphaserquiz@hotmail.com (R.S.); paulaims@gmail.com (P.S.); richeledbq@gmail.com (R.O.); cisteana@yahoo.com.br (A.O.); hugo-alexandre@uol.com.br (H.R.); 2 Department of Nutrition, Federal University of Rio Grande do Norte, Natal, RN, 59072-970, Brazil; Email: aharaujomorais@gmail.com; 3 Department of Cellular Biology and Genetic, Federal University of Rio Grande do Norte, Natal, RN, 59072-970, Brazil; Email: afuchoa2003@yahoo.com.br

**Keywords:** HeLa, Lectin, antitumor, marine sponge, *Cinachyrella apion*

## Abstract

Cancer represents a set of more than 100 diseases, including malignant tumors from different locations. Strategies inducing differentiation have had limited success in the treatment of established cancers. Marine sponges are a biological reservoir of bioactive molecules, especially lectins. Several animal and plant lectins were purified with antitumor activity, mitogenic, anti-inflammatory and antiviral, but there are few reports in the literature describing the mechanism of action of lectins purified from marine sponges to induce apoptosis in human tumor cells. In this work, a lectin purified from the marine sponge *Cinachyrella apion* (CaL) was evaluated with respect to its hemolytic, cytotoxic and antiproliferative properties, besides the ability to induce cell death in tumor cells. The antiproliferative activity of CaL was tested against HeLa, PC3 and 3T3 cell lines, with highest growth inhibition for HeLa, reducing cell growth at a dose dependent manner (0.5–10 µg/mL). Hemolytic activity and toxicity against peripheral blood cells were tested using the concentration of IC_50_ (10 µg/mL) for both trials and twice the IC_50_ for analysis in flow cytometry, indicating that CaL is not toxic to these cells. To assess the mechanism of cell death caused by CaL in HeLa cells, we performed flow cytometry and western blotting. Results showed that lectin probably induces cell death by apoptosis activation by pro-apoptotic protein Bax, promoting mitochondrial membrane permeabilization, cell cycle arrest in S phase and acting as both dependent and/or independent of caspases pathway. These results indicate the potential of CaL in studies of medicine for treating cancer.

## 1. Introduction

Cancer develops due to failures in the mechanisms that normally control cell growth and proliferation. Therefore, losses in the regulation of these cells are, in most cases, caused by genetic damage [1]. Cervical cancer, or cervix cancer, stand out among female genital tract neoplasms, the second most common cancer among women worldwide. With approximately 500,000 new cases per year worldwide, cervical cancer is responsible for the deaths of approximately 230,000 women per year. Conventional cancer treatment can be done in several ways: surgery, radiotherapy, chemotherapy, or in some cases, it is necessary to combine more than one method for treating the cancer. Several distinct biological strategies might prove effective in eliminating established tumors or preventing the maintenance of its progression. The most obvious are designed to induce cancer cell death via apoptosis [2]. Because of the high specificity of interactions with carbohydrates, lectins can serve as marker molecules to specific tumor cell glycoconjugates. In addition, they can be conjugated to a range of carrier agents, acting specifically in malignant cells. In marine invertebrates, most lectins found belong to the family of calcium dependent lectins (C-type lectins), obtained from organisms of different phyla: arthropoda (*Tachypleus tridentatus*), mollusca (*Mytilus edulis*), echinodermata (*Anthocidaris crassispina*, *Cucumaria echinata*) and others [3,4,5,6]. There are few studies in the literature linking properties of lectins from marine sponges with cytotoxic effects or induction of apoptosis in malignant cell lines [7,8,9,10,11]. Our research group purified and characterized a lectin from the marine sponge *Cliona varians* (CvL) [12], with potential antitumor activity on K562 cells (chronic myelogenous leukemia). After the induction of cell death by CvL, the appearance of nuclei with different levels of chromatin condensation and nuclear fragmentation was observed, as well as quantification of apoptotic cells by flow cytometry analysis (43 ± 5% of the total cell population in the apoptotic stage, *p* < 0.05), triggering the release of cathepsin B of vesicular compartments within the cytoplasm with subsequent translocation into the nucleus, without affecting cell viability of normal lymphocytes from human peripheral blood at the same concentrations tested. We have recently purified and characterized a lectin from the marine sponge *Cinachyrella apion*, denominated CaL, which presents strong hemagglutinating activity with preference for papainized type A erythrocytes [6]. The hemagglutinating activity is independent of bivalent ions, and it was strongly inhibited by disaccharide lactose. CaL was heat-stable between 0 and 60 °C and pH-stable. The lectin has a molecular mass of 124 kDa, consisting of eight subunits of 15.5 kDa, assembled by non-covalent interactions. CaL also agglutinated *Leishmania chagasi* promastigotes, and this activity was arrested by lactose. In this work, we show that CaL inhibits proliferation of cultured tumor cell lineage by the induction of cell death.

## 2. Results

### 2.1. Effects of CaL on Cell Proliferation of Tumor Cells Lines

**Figure 1:**
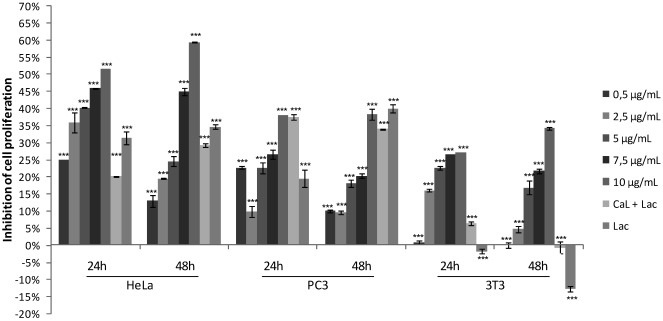
Effect of CaL on viability of cell lines PC3, 3T3 and HeLa. The cytotoxicity of CaL on the tumor lines PC3 and HeLa and against the normal mouse fibroblast 3T3 line was performed by MTT reduction assay. The test cells were treated with different concentrations of CaL (0.5–10 µg/mL) for 24 and 48 h of culture in microplates. CaL (10 µg/mL) incubated with specific inhibitor lactose (0.1 M) was used. The viability of cells treated with CaL was expressed as a percentage of the viability of untreated control cells. Results represent the mean ° SD (standard deviation) of three experiments run in three replicates. *** *p* < 0.001 compared to control (Student-Newman-Keuls test).

**Figure 2:**
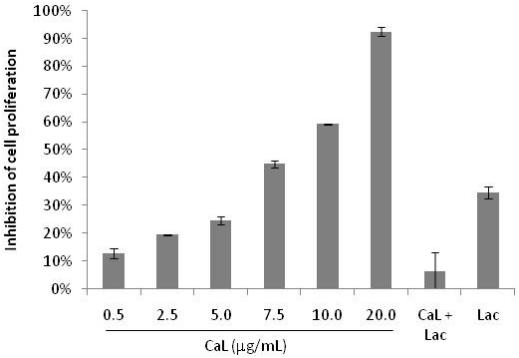
Cytotoxicity of CaL on HeLa tumor strain. HeLa cells were incubated with different concentrations of CaL until twice the IC_50_ (20 µg/mL) for 48 h, and the cell proliferation was evaluated and compared with untreated control cells. CaL (20 µg/mL) pre-incubated with lactose and only lactose was also tested. Results represent the mean ° SD of three experiments run in three replicates.

The cytotoxicity of CaL to HeLa, PC3 and 3T3 cells was investigated after an incubation period of 24 and 48 h using the colorimetric MTT assay (Figure 1). HeLa and PC3 cell proliferation were inhibited in a dose-dependent manner in response to increasing concentrations of CaL (0.5–10 µg/mL). CaL also presented toxicity against 3T3 cells, although it had low significance in comparison to the other cell lines tested. HeLa cells had a greater inhibition rate after CaL treatment, so this lineage was used in further tests. The 50% inhibition (IC_50_) was obtained with a concentration of 10 µg/mL of CaL, confirmed with an independent experiment using 20 µg/mL of CaL as a final concentration (Figure 2), a dose that inhibited around 95% of HeLa cell proliferation. Pre-incubation of CaL with lactose reduced significantly its antiproliferative activity on the HeLa cell (Figure 1 and Figure 2), indicating that there may be a close link between the lectin-active domain and its antiproliferative activity.

### 2.2. Cytotoxicity on Human Peripheral Blood Cells and Hemolytic Activity of CaL

**Figure 3:**
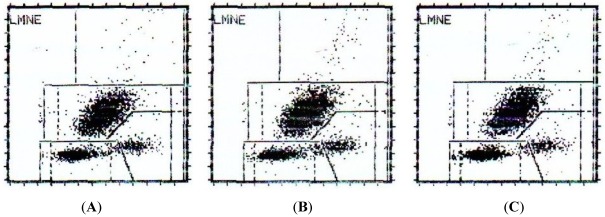
Flow Cytometry of human peripheral blood in the presence and absence of CaL. (**A**) Human peripheral blood in absence of CaL; (**B**) Human peripheral blood in presence of CaL, 10 µg/mL (80.65 nM); (**C**) Human peripheral blood in presence of CaL, 20 µg/mL (161.29 nM).

**Figure 4:**
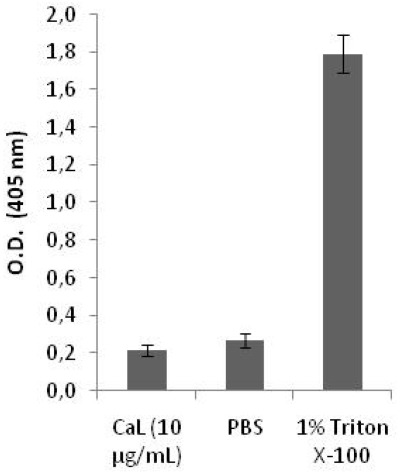
Flow Cytometry of human peripheral blood in the presence and absence of CaL. Evaluation of hemolytic effect of CaL on human red blood cells. Negative and positive controls were used: phosphate buffered saline (PBS) and 1% Triton X-100, respectively. Results represent the mean ° SD of three experiments run in triplicate.

To test the CaL toxicity against normal cells, the lectin was incubated with erythrocytes and peripheral blood cells, using bovine serum albumin as control. CaL did not show cytotoxicity against human peripheral blood cells when evaluated in blood cell counter and flow cytometry (Figure 3) based on two concentrations: 10 µg/mL and 20 µg/mL (corresponding to one and two times its IC_50_, respectively). There was also no hemolytic activity for CaL using IC_50_ concentration, as seen in the hemolytic assay performed in a 96-well plate (Figure 4).

### 2.3. Nuclear Morphological Changes Induced by CaL in HeLa Cells

**Figure 5:**
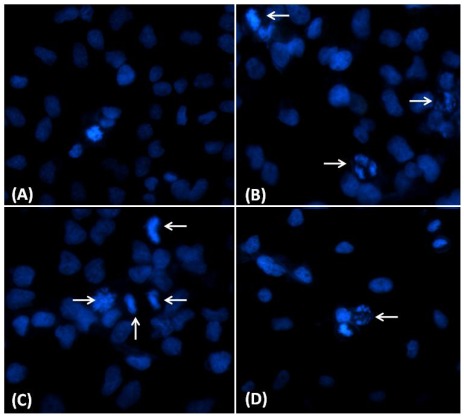
Micrograph of HeLa cells treated with CaL. HeLa cells were incubated with 10 µg/mL (80.65 nM) CaL 24 h and labeled with DAPI to show nuclear morphology. (**A**) Control HeLa cells, without CaL; (**B–D**) HeLa cells treated with CaL, showing nuclear morphological changes such as pyknosis and fragmentation (arrows).

Nuclear morphological changes were observed by DAPI staining. In the control group, HeLa cells were round in shape and stained homogeneously (Figure 5A). After 24 h treatment with CaL, blebbing nuclei, picnotic bodies, morphological alterations and granular apoptotic bodies appeared (Figure 5B–D). Markable morphological alterations, including membrane blebbing and nuclear condensation, suggest CaL induces apoptosis in HeLa cells.

### 2.4. Balance between Apoptosis and Necrosis by Double Staining Annexin V-FITC/PI and Cell Cycle Analysis

**Figure 6:**
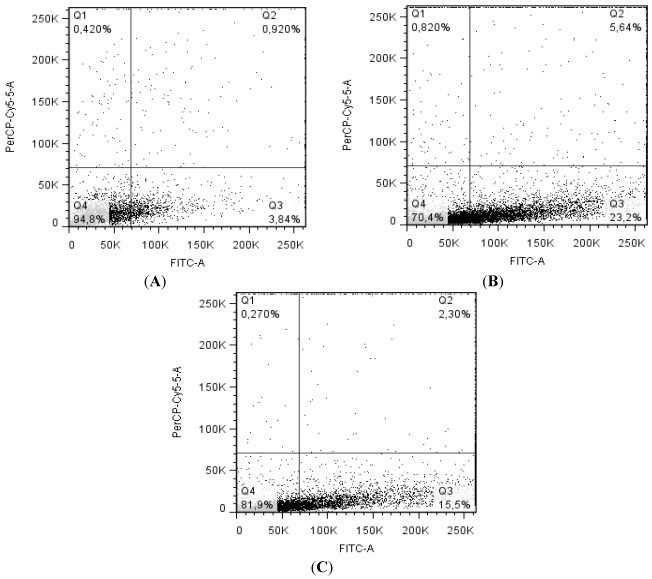
Flow Cytometry of HeLa cells treated with annexin V/PI and CaL. The cells were maintained in the presence of 10 µg/mL lectin (with or without Z-VAD-FMK) for 24 h, stained with FITC-annexin V/PI and analyzed by FACScan flow cytometer marked for apoptosis/necrosis. Q1: Annexin V negative/PI positive; Q2: Annexin V/PI positive; Q3: Annexin V positive/PI negative; Q4: Annexin V/PI negative. (**A**) Control cells without the presence of CaL; (**B**) Cells incubated with 80.65 nM CaL; (**C**) Cells incubated with 80.65 nM CaL along with ZVAD-FMK. One representative chart of two independent experiments for each sample is presented. The analysis of data from flow Cytometry was performed using the FlowJo software [39].

**Figure 7:**
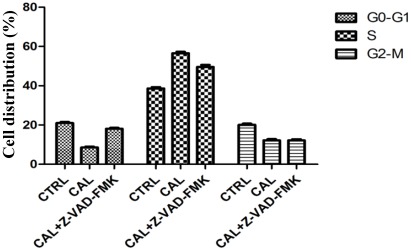
Evaluation of cell-cycle arrest after exposure to CaL. After 24 h of treatment with 80.65 nM CaL (with or without Z-VAD-FMK), HeLa cells were fixed, treated with RNase, stained with PI and analyzed by flow cytometry for assessment of cell cycle distribution. Results represent the mean ° SD of three experiments run in triplicate. Data analyses for cell cycle arrest were performed using the FlowJo software [39].

To further characterize if CaL-induced HeLa cell death was accomplished by apoptosis or necrosis, the ratios of apoptosis and necrosis in cells were analyzed by flow cytometry after staining of cells with annexin V-FITC/PI. Exposure of 10 µg/mL CaL significantly (*p* < 0.001) increased the percentage of cell death in 23.2% of cells marked only with annexin V (Figure 6A,B), indicating an early stage of apoptosis, compared to control, with less than 4% of HeLa cells initiating apoptosis (An+/PI^−^). To evaluate the involvement of caspases in CaL-induced cell death, Z-VAD-FMK (pan-caspase inhibitor) was applied. After 24 h incubation with CaL, Z-VAD-FMK reduced in 7.7% cell death (Figure 6C), indicating more than one pathway to apoptosis induced by CaL, both dependent and/or independent of caspases. To minimize the adverse effects of physiological situations stressful to cells, arrest of cell cycle progression can be provided quickly after exposure to stress stimulus. Thus, the effect of the lectin on the distribution of cells in different phases of the cell cycle was analyzed by flow cytometry after staining of cells with PI (Figure 7). Untreated control HeLa cells exhibited normal cell cycle characteristics (G_1_/G_0_ e G_2_/M phases). Incubation of cells with CaL for 24 h induced cell cycle arrest in S phase, with 57.6% of cells in this phase. The presence of Z-VAD-FMK was not able to interfere with cell cycle arrest. Altogether, these results indicate CaL was able to induce apoptotic cell death in HeLa cells.

### 2.5. CaL Effects in Activation of Proteins Related to Apoptosis in HeLa Cells

**Figure 8:**
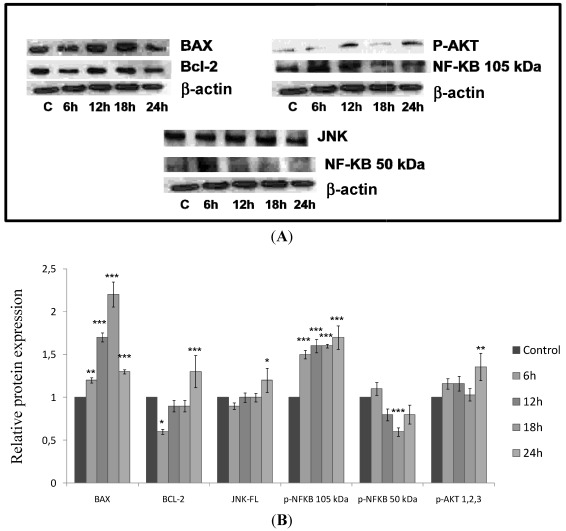
Immunoblotting of proteins involved in cell death by apoptosis. (**A**) Protein banding derived from Western Blotting; (**B**) Graphic expression of BAX, Bcl-2, JNK, p-NFκB (105 kDa and 50 kDa) and p-AKT. One representative immunoblot of three independent experiments is presented. *** *p* < 0.001, ** *p* < 0.01, * *p* < 0.05, compared to control (Student-Newman-Keuls test).

To determine the key proteins involved in inducing cell death in HeLa cells, the Western blotting technique was used. A fixed concentration of CaL (80.65 nM) was incubated with HeLa cells at different incubation times (control, 6 h, 12 h, 18 h and 24 h) (Figure 8). There was a considerable increase in expression of Bax (pro-apoptotic protein belonging to the Bcl-2 family) and NF-κB (transcription factor of nuclear factor κB) in the form of 105 kDa precursor where this increase was gradual, *i.e.*, in a time-dependent manner. Despite the increase in BAX, anti-apoptotic protein Bcl-2 showed no significant change in expression compared to control (Figure 8B). Western blotting of other proteins related to cell death by apoptosis was also assessed, such as JNK, p-AKT and NF-κB of 50 kDa (active form), which showed no change in activation of p-AKT in the presence of CaL. However, there was a considerable reduction in the active form of NF-κB (Figure 8A,B) and a slight increase in JNK activation, over the 24 h period. These data, together with results obtained in flow cytometry, indicate the toxic effect of CaL in HeLa cells, suggesting apoptotic cell death through activation of Bax, a pro-apoptotic protein, with mitochondrial membrane permeabilization, acting probably in an intrinsic pathway both dependently and/or independently of caspases.

## 3. Discussions

After a quarter century of advances, cancer research had generated a complex and rich source of knowledge, showing cancer as a disease involving dynamic changes in genome [2]. Several lines of evidence indicate that tumorigenesis in humans is a multistep process, and these steps reflect genetic alterations that lead to the progressive transformation of normal human cells to highly malignant derivatives [13].

In recent decades, large numbers of studies have unraveled the pathways of signal transduction in the control of cell death and the molecular machinery responsible for these processes, leading to numerous opportunities for pharmacological intervention and drug design [14]. Among new drugs used in cancer therapy with the potential to interfere with the regulation mechanism of tumor cell growth, natural products have drawn attention as important sources of chemotherapeutic agents and/or chemical and structural models for the development of a multitude of compounds [2,15,16,17,18]. Lectins are a class of proteins widely distributed among living organisms, and have attracted attention as potential activators of cell death in tumor tissues by triggering apoptotic signaling cascades [19,20,21,22,23,24,25,26].

The purification procedure of CaL was reproduced according to Medeiros *et al*. (2010) [27]. Antiproliferative activity of CaL on cell lines HeLa, PC3 and 3T3 was evaluated. CaL was able to induce growth inhibition in all cell lines tested, especially on HeLa cells, in a dose dependent manner, with IC_50_ of 10 µg/mL (80.65 nM). To prove that the cytotoxic effect of the lectin was not time dependent, tumor cells were exposed, in independent experiments up to 48 h of treatment with CaL, keeping the profile of cytotoxicity to the cells tested in both test times. Similar results were found for the fungus *Agrocybe aegerita* lectin (AAL) and the sponge *Haliclona cratera* with IC_50_ of 10 µg/mL and 9 µg/mL, respectively, against adenocarcinoma cells [10,28]. However, CaL had a lower IC_50_ compared with the lectin *Astragalus mongholicus* root (40 µg/mL) [29]. In addition to incubation of cells with CaL, its specific inhibitor (lactose 0.1 M) was tested, either alone or in association with lectin, to assess its antiproliferative activity. There are no reports of antiproliferative activity induced by lactose, so this data is particularly important, since lactose was able to reduce proliferation in approximately 40% of tumor cells, and stimulate the proliferation of 3T3 cells. 

CaL showed no cytotoxic activity, even if incubated with twice its IC_50_ (20 µg/mL or 161.3 nM) against human erythrocytes or peripheral blood cells. Dresh *et al*. (2005) [6] analyzed the hemolytic and lectin activity of twenty extracts of sponges from the Atlantic coast of Brazil and found that only two of these extracts showed hemolytic activity, highlighting the potential of lectins as potential candidates for biopharmaceuticals. These results are particularly important, since many traditional chemotherapeutic agents exhibit severe toxicity against normal cells, causing undesirable side effects and thus limiting their application in the clinical field. For this reason there is a clear need for new agents with different mechanisms of action that can be used in direct treatment of these diseases and/or as adjuvant therapy in improving cancer outcomes [15,30].

The visualization of the morphological changes of the HeLa cell line after exposure to CaL was observed by fluorescence microscopy, marked with the dye DAPI, to indicate the activation of cell death by apoptosis. Although these results present strong evidence that the apoptotic pathway is activated, other tests for the analysis of molecular characters are needed to corroborate the results obtained in microscopy. Other studies used the technique of labeling with DAPI for visualization of morphological changes in tumor cells [29,31,32,33,34].

The ability to induce apoptosis in CaL was visualized by flow cytometry with annexin V-FITC/PI markup. After 24 h, it was possible to evaluate the induction of apoptosis in HeLa cells by the purified lectin, with approximately 23% of cells stained only for annexin V. In order to identify the cell death pathway promoted by the lectin, the cells were incubated with CaL and a general cysteine-protease inhibitor capable of binding irreversibly to the catalytic sites of caspases (Z-VAD-FMK). Flow cytometry results indicated that the presence of Z-VAD-FMK reduced the percentage of cells undergoing apoptosis in 7.7%, *i.e.*, CaL probably induces cell death both dependently and/or independently of caspase activity. Miyoshy and colleagues found the effect of rice bran agglutinin (RBA), wheat germ agglutinin (WGA) and *Viscum album* agglutinin (VAA) in the induction of apoptosis in U937 cells, proving that RBA induces chromatin condensation, externalization of phosphatidylserine, visualized flow cytometry and DNA fragmentation, and suggests that the mechanism of cell death promoted by RBA is similar to WGA, but different for VAA [23].

Many anti-cancer agents and modifiers of DNA arrest cells in the G0/G1 phase, S or G2/M by inducing apoptotic cell death [35]. Flow cytometry analysis indicated cell cycle arrest in the S phase (57% of cells) in HeLa cells after incubation with CaL for 24 h, both in the presence or absence of cysteine-protease inhibitor Z-VAD-FMK. Cell cycle arrest in S phase is not a common event for lectins. Flow cytometry indicates that fungus *Volvariella volvacea* lectin (VVL) holds cell proliferation by blocking cell cycle progression in G2/M phase [36]. A rare reference to this effect was reported for the lectin purified from the roots of *Astragalus mongholicus* (AMML), which led to imprisonment of HeLa cells in S phase after 24 h of exposure, to a lesser degree (34–39% arrest) than CaL and a higher concentration range (20–40 µg/mL AMML) than used with CaL [29]. Imprisonment in S phase was observed in some cell lines treated with sodium ascorbate [37] and an inhibitor of tyrosine kinase Jak-AG490 cells [38]. However, the mechanism responsible for this effect remains unclear.

CaL findings indicate that, by Western Blot analysis, increased levels of Bax can be observed up to 18 h, decreasing thereafter in 24 h, while not altering the expression levels of Bcl-2. These data suggest that probably CaL induced mitochondrial membrane permeabilization in HeLa cells, probably activating the apoptotic intrinsic pathway. The levels of anti-apoptotic protein NF-κB in its inactive form increased progressively with time of exposure to CaL, whereas the activation of Akt remained unchanged. These events promote the activation of apoptosis, since these proteins are involved in the activation of anti-apoptotic members of the Bcl-2 family and IAPS, among other molecules that promote cell survival. The route of apoptotic cell death activated by JNK was not discarded, despite the changes in JNK activation being seen in only 24 h. Future studies may clarify the role of this protein in the antiproliferative mechanism of CaL.

Thus, one can suggest that CaL operates in HeLa cells inducing apoptosis through the activation (not exclusive) of the mitochondrial intrinsic pathway, acting both dependently and/or independently of caspases stimulating mitochondrial membrane permeability and hence, the release of proteins such as cytochrome *c*, AIF and/or EndoG. Several models of cell death involving the participation of initiator/executor caspases, as well as apoptotic cell death independent of caspases have been established, but further studies regarding the participation of other proteins related to this type of cell death are needed to determine the mechanism of action of CaL in human adenocarcinoma cell (HeLa) apoptosis.

## 4. Experimental Section

### 4.1. Materials

Papain and bovine trypsin were purchased from Sigma Chemical Co. (St. Louis, MO, USA). Human erythrocytes type A, B and O were donated by the Blood Bank, Hemocentro, Natal, Brazil. Rabbit polyclonal antibodies to human Bax and Bcl-2, rabbit anti-cleaved caspase-3 monoclonal antibody and secondary antibody produced in conjunction with peroxidase goat anti-rabbit were obtained from Cell Signaling Technology (Beverly, MA, USA). Rabbit anti-JNK, anti-p-Akt and anti-NF-κB polyclonal antibodies and peroxidase-conjugated secondary antibody obtained from goat anti-rabbit were obtained from Santa Cruz Biotechnology (Santa Cruz, CA, USA).

### 4.2. Preparation of Marine Sponge Cinachyrella apion Lectin (CaL)

Specimens of the marine sponge *Cinachyrella apion* were collected on the coast of Santa Rita, Extremoz, RN, Brazil. After collecting, they were transported in ice to the laboratory and stored at −20 °C until use. The species was identified by Eduardo Carlos Meduna Hajdu and deposited (Number of collection MNRJ 10142) in Museu Nacional, Rio de Janeiro, Brazil. Lectin from the marine sponge *Cinachyrella apion* (CaL) was purified essentially as previously described [27]. In short, the lectin was extracted with Tris-HCl buffer, fractionated by acetone precipitation and purified by immunoaffinity and fast protein liquid (FPLC-AKTA purifier) chromatographies.

### 4.3. Cell Culture

HeLa, a human cervical adenocarcinoma cell line, 3T3, an immortalized mouse fibroblast line and PC3, a human prostate adenocarcinoma were obtained from American Type Culture Collection (ATCC, Rockville, MD, USA). Cell lines HeLa and 3T3 were grown in DMEM (Dulbecco’s Modified Eagle’s Medium), supplemented with 10% fetal calf serum, adding streptomycin (5000 mg/mL)/penicillin (5000 IU). PC3 cells were grown in a RPMI-1640 medium, supplemented with 10% fetal bovine serum, and treated with streptomycin (5000 mg/mL)/penicillin (5000 IU), kept in a sterile environment at 37 °C with 5% CO_2_ in a humidified atmosphere.

### 4.4. Cell Growth Inhibition Assay

HeLa cells were dispensed in 96-well flat-bottomed microtiter plates (TPP products, Switzerland) at a density of 5 × 10^3^ cells/well. Cells were incubated for 24 and 48 h with CaL at given concentrations. The effects of CaL on cell proliferation were determined using the MTT assay with a plate reader. To assess the effect of carbohydrates on CaL-induced HeLa cell death, the MTT assay was determined as above except that lectin was pre-incubated for an hour with lactose 100 mM (CaL specific inhibitor). The test was performed in triplicate. The measurement of cell proliferation inhibition was carried out in comparison with control containing untreated cells with the lectin purified as follows:


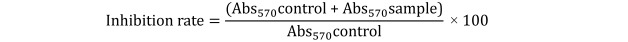


### 4.5. Cytotoxicity of Human Peripheral Blood Cells and Hemolytic Activity *in Vitro*

BSA was used as control at different concentrations (5 to 50 µg/mL) for 15 min at 37 °C to evaluate possible hematological changes. The cell viability was assessed by blood cell counter (CHCELL 6019-Laborlab). To assess the hemolytic effect of CaL, human erythrocytes were separated from plasma by sedimentation and washed three times with Tris-HCl pH 7.4 containing 0.01 M NaCl 0.15 M. The same buffer was used to prepare a suspension of 1% (v/v) red blood cells and solubilize the samples. 100 µL of suspension of red blood cells in 1.5 mL tubes were incubated with 100 µL of sample for 60 min at room temperature. References to 100% and 0% hemolysis were made by incubating a 100 µL suspension of red cells with 100 µL Triton X-100 1% (v/v) or 100 µL of Tris buffer, respectively. After incubation, the tubes were centrifuged at 3000 × g for 2 min and 100 µL aliquots of the supernatants were transferred to microtiter plates of 96 wells and analyzed at 405 nm.

### 4.6. Evaluation of Indicators of Apoptosis by Incubation with DAPI

HeLa cell line was seeded on 13 mm circular coverslips in a 24-well plate (35.55 × 10^4^ cells/well). After 45 min at 37 °C, DMEM medium supplemented with 10% FBS was added in a humidified atmosphere of 5% CO_2_, to a final volume of 1 mL. 24 h later, the medium was removed and the cells were deprived for 24 h with medium without serum. After treatment with medium supplemented with CaL solubilized at a concentration of 10 µg/mL (80.65 nM), cells were washed with cold phosphate buffer (PBS), fixed with 4% paraformaldehyde for 20 min and permeabilized in 0.1% Triton X-100 for about 20 min. Subsequently, cells were washed again with PBS and incubated with DAPI (4′,6-diamidino-2-phenylindole) at a concentration of 1 mg/mL for 30 min, protected from light at room temperature. Cells were visualized by fluorescence microscopy (fluorescence microscope OLYMPUS BX41) using the fluorescence filter 330–380 nm.

### 4.7. Annexin V-FITC/PI Double Staining and Analysis by Flow Cytometry

To evaluate the effects of CaL on cell death, the FITC/annexin V Apoptosis Kit with Dead Cell Annexin FITC and PI, for Flow Cytometry (Invitrogen, Catalog No. V13242), was used. Cells were grown in 6-well plates until they reached confluence of 2 × 10^5^ cells/mL with medium without serum and stimulated to exit G_0_ in the presence of purified lectin solubilized in DMEM, supplemented with 10% FBS for 24 h. In addition, a negative control was prepared without the presence of CaL, and the action of CaL incubated with general caspase inhibitor Z-VAD-FMK (carbobenzoxy-valyl-alanyl-aspartyl-[*O*-methyl]-fluoromethylketone) (0.02 mM) was also tested to confirm the mechanism of action by which the lectin induces cell death. After exposure to a concentration of 10 µg/mL (80.65 nM) of CaL for 24 h, HeLa cells were trypsinized, collected and washed with cold PBS. The supernatant was discarded and the cells were resuspended in 200 µL of 1X Binding Buffer. 5 µL of annexin V-FITC and 1 µL of PI solution (100 µg/mL) were added in a 100 µL cell suspension. The cells were incubated for 15 min under room temperature and kept under light protection. After the incubation period, 400 µL of binding buffer for annexin V 1X was added and cells were analyzed by flow cytometry (flow cytometer FASCANTO II, BD Biosciences), measuring the fluorescence emission at 530–575 nm for annexin V and 630–22 nm for PI. For data analysis, FlowJo software [39] was used.

### 4.8. Cell Cycle Analysis

HeLa cells were washed with cold PBS and the supernatant was discarded. The pellet with cells was then incubated with 2% paraformaldehyde, washed with cold PBS and permeabilized with 0.01% saponin for 15 min. After this procedure, the cells were incubated with 10 µL of RNase (4 mg/mL) at 37 °C for 30 min. 5 µL of PI solution (25 mg/mL) along with 200 µL of cold PBS to cells were added and taken to the flow cytometer for analysis of cell cycle arrest (630–22 nm). The percentage of apoptotic cells was determined every 20,000 events and graphs obtained in the experiment represent data from three independent experiments. FlowJo software [39] was used for data analysis.

### 4.9. Western Blotting

HeLa cells were plated at a concentration of 9.6 × 10^5^ cells in 75 mL sterile bottles and incubated for 24 h for adhesion. A fixed concentration of 10 µg/mL of CaL was added to cells at different times of incubation (0 h, 6 h, 12 h, 18 h and 24 h), washed with cold PBS and removed with 200 µL of lysis buffer [50 mM Tris-HCl (pH 7.4), 1% Tween 20, 0.25% sodium deoxycholate, 150 mM NaCl, 1 mM EGTA, 1 mM Na_3_VO_4_, 1 mM NaF and the following protease inhibitors for 2 h on ice: 1 µg/mL aprotinin, 10 µg/mL and 1 mM leupeptin fluoride of phenylmethanesulfonyl]. Total protein extracts were obtained and a polyacrylamide gel electrophoresis in the presence of SDS was carried out following an established methodology [40]. Protein extracts were resolved and electrophoretically transferred to a polyvinylidene fluoride (PVDF) membrane (Millipore, Bedford, MA, USA) [35]. After transfer, membranes were blocked for non-specific sites with blocking buffer [1% skim milk or 2% fetal bovine serum (BSA) in Tris-buffered saline (TBS) with 0.05% Tween 20 (TBST)], remaining in this solution for one hour, and then incubated for about 12 h at 4 °C with appropriate primary antibody diluted in blocking buffer at a ratio of 1:1000. After washing in TBST, membranes were incubated with anti-rabbit secondary antibody conjugated with peroxidase, diluted 1:2000 in blocking buffer for 1 h. The detection was performed using chemiluminescence [41].

### 4.10. Statistical Analysis

All data represent at least three independent experiments and were expressed as mean ± SD of triplicates, except were otherwise noted. Differences between groups were compared by the Student-Newman-Keuls or Tukey test, used to show some similarities found by ANOVA. Differences were considered significant when *p* value was less than 0.05. Statistical data were analyzed by GraphPad InStat 3.05 [42].

## 5. Conclusion

A lectin was purified from the sponge *Cinachyrella apion* (CaL) and showed preferential binding activity for type A erythrocytes, treated with papain, despite the presence of divalent ions. The hemagglutinating activity of CaL was strongly inhibited by disaccharide lactose. CaL showed no hemolytic or toxicity activity against peripheral blood cells at concentrations of IC_50._ In addition, this lectin showed high antiproliferative potential against tumor cell lines tested, especially in HeLa cells, acting in a dose-dependent manner. These results indicate that CaL induces apoptotic cell death in HeLa cells, probably by activating the mitochondrial intrinsic pathway, by both pathways, dependent and/or independent of caspases, stimulating mitochondrial membrane permeabilization and promoting the release of cytochrome c, AIF and/or endonuclease G.
